# The impact of a park refurbishment in a low socioeconomic area on physical activity: a cost-effectiveness study

**DOI:** 10.1186/s12966-019-0786-5

**Published:** 2019-03-08

**Authors:** Anita Lal, Marj Moodie, Gavin Abbott, Alison Carver, Jo Salmon, Billie Giles-Corti, Anna Timperio, Jenny Veitch

**Affiliations:** 1 Deakin University, Geelong, Australia, Deakin Health Economics, Centre for Population Health Research, Locked Bag 20000, Geelong, VIC 3220 Australia; 2 Institute for Physical Activity and Nutrition (IPAN), School of Exercise and Nutrition Sciences, Deakin University, Geelong, Australia; 3 Mary MacKillop, Australian Catholic University, Institute for Health Research, Melbourne, Australia; 4 Centre for Urban Research, RMIT University, Melbourne, Australia

**Keywords:** Physical activity, Park, Playground, Play-scape, Children, Socioeconomic, Cost-effectiveness

## Abstract

**Background:**

Physical inactivity is the fourth highest cause of death globally and is a major contributor to increases in healthcare expenditure. Improving public open spaces such as parks in areas of low socio-economic position (SEP) may increase recreational physical activity in disadvantaged populations. We assessed the cost-effectiveness of the installation of a play-space in a large metropolitan park in a low socioeconomic area based on changes in physical activity.

**Methods:**

Observational data of visitor counts and activities undertaken in the park before the installation of the new play-scape (T1), at two months (T2) and 14 months post-installation (T3) were obtained for the intervention and a control park (with no refurbishment) located in a high SEP metropolitan area. Observed sitting, standing, and moderate and vigorous-intensity physical activity were converted to yearly MET-h according to age. Costs of the play-scape and ongoing maintenance were obtained from the organisation managing the refurbishment. The incremental cost-effectiveness ratio (ICER) (ratio of incremental cost to incremental effect) was calculated based on the incremental increase in MET-h from T1 to T3 assuming a 20-year lifetime of the play-scape. Observation counts combining moderate and vigorous activity were used in the sensitivity analysis.

**Results:**

When compared with T1, at T3 the new play-scape resulted in an overall incremental net gain of 114,114 MET-h (95% UI: 80,476 − 146,096) compared with the control park and an incremental cost effectiveness ratio (or cost per MET-h gained per park visitor) of AUD $0.58 (95% UI: $0.44–$0.80).

The sensitivity analysis combining moderate and vigorous activity into one category showed an increase in estimated incremental MET-h of 118,190 (95% CI: 83,528 − 149,583) and a lower incremental cost per MET-h gained of AUD $0.56 (95% UI: $0.43–$0.77).

**Conclusions:**

Using a benchmark of cost-effectiveness for physical activity interventions of AUD $0.60–$1.30, this study suggests that the installation of a play-scape located in a low SEP area is cost-effective based on its potential to facilitate increases in MET-h. It provides much needed preliminary evidence and requires replication elsewhere.

**Electronic supplementary material:**

The online version of this article (10.1186/s12966-019-0786-5) contains supplementary material, which is available to authorized users.

## Introduction

Physical inactivity is the fourth highest cause of death globally [1] and is a major contributor to increases in healthcare expenditure [2]. In Australia, only one-third of children undertake the recommended 60 min of physical activity every day [3], and just 45% adults engage in 150 min/week of at least moderate-intensity physical activity [4]. These low prevalence rates are consistent with many other high-income countries globally [5]. There is also consistent evidence of a lower prevalence of moderate- to vigorous-intensity physical activity among adults of lower socio-economic position (SEP) in high income countries [6–8].

Public open spaces, including parks, provide opportunities for physical activity across a range of diverse population groups [9]. Parks with more amenities have been shown to increase use and encourage physical activity [10, 11]. Yet compared with high SEP neighbourhoods, parks in low SEP areas have been shown to have fewer amenities likely to encourage physical activity [12] and tend to be of lower quality in terms of amenities and aesthetics [13]. It is therefore plausible that improving parks in low SEP areas could help to increase physical activity among disadvantaged populations.

Governments – particularly local government - make significant financial investments to improve and maintain parks. While previous studies have examined changes in park visitation and park-based physical activity after park refurbishment [14–16], there is very little international evidence about whether park refurbishment is an efficient and cost-effective way to allocate resources to increase physical activity [17–19]. Such information is critical given government budgets are often tightly restricted with competing priorities for funding. A limited number of studies in the US have examined the cost-effectiveness of parks including new playgrounds and outdoor exercise equipment and have found that cost-effectiveness ranged from USD 0.105–2.66 [14, 17, 20].

A recent Australian study demonstrated a 128% increase in park visitors observed engaging in physical activity following the installation of a play-scape designed to bring children and accompanying adults back to nature, relative to a control park [21]. The current study aimed to assess the cost-effectiveness of this play-scape installation.

## Methods

The Recording and EValuating Activity in a Modified Park (REVAMP) study was a natural experiment designed to evaluate the impact of the installation of a play-scape, a play area designed with the intent of bringing children and accompanying adults back to nature, in a large metropolitan park (Brimbank Park) in Melbourne, Australia. The study was registered in the International Randomised International Standard Randomised Controlled Trials Number registry (ISRCTN50745547) and the protocol and main outcomes have been published [21, 22].

### Intervention

The 329 ha intervention park was located 28 km north-west of Melbourne’s Central Business District. It is located in a municipality of extreme socioeconomic disadvantage, with one of the highest percentage of low income families in the state of Victoria [23].

The refurbishment involved the installation of a new play-scape including a large 360 degree swing, traditional swing set, maze, rockers, sandpit, nature play area, climbing equipment, landscaping, and various sculptures. The play-scape was designed to be accessible for children with disabilities and to encourage visitors to connect with both the natural environment and the indigenous cultural heritage of the region. The play-scape area was originally an open space area with no features or amenities. The refurbishment of the park occurred between September 2013 and February 2014.

### Control

The control park (Westerfolds Park, 120 ha) was located 22 km east of Melbourne’s Central Business District in a high SEP area. The investigators were unable to find a similar sized park in a low SEP area that was not undergoing renovations during the study period, however the selected park had similar infrastructure and settings for being active, such as paths for walking or cycling, open space areas and a single adventure style playground. In addition, both parks had other similar amenities such as toilets, car-parking, a variety of picnic shelters, tables and barbeque areas. A more detailed description of the features within the two parks can be found elsewhere [24]. It is recognised that it may be challenging to identify a perfectly matched control site and a compromise may be necessary [25, 26].

#### Assessment of benefits

##### Primary outcome

The outcome of interest was a change in the number of visitors across three age groups (1–12 years, 13–20 years and > 21 years) observed engaging in physical activity, measured in metabolic equivalent hours (MET-h). Observational data of park visitors were recorded within both parks over eight days, including four weekdays and four weekend days using a modified version of the System for Observing Play and Recreation in Communities (SOPARC) [21, 27]. As described previously [28], observations were conducted at hourly intervals between 7.30 am-4.30 pm on weekdays and between 8.30 am-4.30 pm on weekend days. Baseline assessments were conducted in April–May 2013 (T1 – pre-park refurbishment), first follow-up measures were conducted in April–May 2014 (T2 – two months after the completion of refurbishment) and second follow-up measures were conducted in April–May 2015 (T3–14 months after the completion of refurbishment). Each data collection phase took place on the same days at both parks and at the same time of the year (autumn) to account for potential seasonal effects.

Research staff recorded each individual in specified target areas according to their estimated age group and the activity they were performing (sitting, standing, and moderate and vigorous-intensity physical activity). The target areas included the new play-scape at the intervention park and the playground at the control park, as well as other areas within the parks such as open spaces and picnic shelters. For the primary analysis, only the counts at the newly installed play-scape area at the intervention park and the playground area at the control park were used, not the total counts across all recorded target areas.

##### Calculation of MET- hours

At each park, an average hourly count of park visitors engaged in each activity was obtained. The counts of people doing activity at a particular moment in time was used as an estimate of the total person hours of that activity during each one hour period (See Additional file 1 for detailed counts of people). For the purpose of this paper, MET values were assigned to observed sitting, standing, and moderate and vigorous-intensity physical activity according to age [29, 30] (Table 1). For each activity, MET values for children aged 6–9 and 10–12 years were averaged to obtain a value for all children aged 12 years and under. Similar calculations were carried out for MET values for the 13–20 year category.

**Table 1:** MET-h values applied to each activity level by age group

	Sitting	Standing	Moderate activity	Vigorous activity	Average of moderate and vigorous activity
Up to 12 years	1.5^1^	1.7	6.0^2^	7.3^3^	6.7
13–20 years	1.5^1^	1.7	6.3^2^	7.7^3^	7.0
20 years and over	1.5	2.3	3.5^4^	7.0^5^	5.3

^1^ Sitting quietly [46]; ^2^ Obstacle/locomotor course-moderate [46]; ^3^ obstacle/locomotor course-vigorous [46]; ^4^ walking for pleasure [30]; ^5^ jogging [30]

To obtain an estimate of annual use, the average counts on weekends and weekdays were multiplied by the number of weekend days and weekdays in a year. It was assumed that park visitation would not occur on days of significant rainfall, therefore to account for the average of 20 days of rain > 10 mm per year in Melbourne, [31] we based our calculations on 49 weeks of the year.

For example, to calculate the total number of MET-h for children, the average counts of children per day engaged in each category of physical activity were multiplied by the number of MET-h according to age. This was then multiplied by the number of days in 49 weeks.

The main analysis used data from baseline (T1) and the final follow up (T3), which took place more than a year after the refurbishment was completed, and was thought to more accurately reflect the average number of visitors to be sustained over time.

### The economic evaluation

A cost-effectiveness analysis was conducted to determine the incremental cost-effectiveness ratio (ICER) per unit increase in MET-h gained, using the ratio of incremental cost to incremental effect between observed park-based physical activity at the play-scape/playground area at T1 vs T3, relative to the control park. ICERs were calculated by determining the increment in MET-h generated per cost of the play-scape, per year.

Table 2 outlines the general methodology of the economic evaluation. The intervention was assumed to be operating in ‘steady state’ at T3 (i.e. running at its full effectiveness potential with typical numbers of visitors) and measured against pre-refurbishment. Discounting in cost-effectiveness analysis allows for fair comparisons of interventions when costs and outcomes occur at different times [32]. After year one, each years’ costs were discounted at a rate of 3%.

**Table 2:** General methods of the economic evaluation

Parameter	Method
Perspective	Local government perspective
Study design	Cost-effectiveness analysis
Monetary unit of measurement	Australian Dollars
Base year	2017
Unit of measurement of outcomes	Metabolic equivalent hours (MET-h) gained
Comparator	Control park, no refurbishment
Discounting	3%

#### Costs

The costs of the play-scape installation and its yearly maintenance, as well as maintenance costs for the control park, were obtained from Parks Victoria, the organisation that managed the refurbishment (Table 3). Refurbishment costs were inflated to 2017 prices using the relevant CPI [33].

**Table 3:** Key model variables

Parameters	Mean values	Data source and assumptions
*Intervention effect estimates*		
Difference in mean observations of children, adolescents and adults per day at T1 and T3^*^	Additional file 1	Veitch et al. [21] Uncertainty analysis used PERT distributions of observations (Additional file 1)
% of people engaged in physical activity from observations of children, adolescents and adults per day at T1 and T3^*^	Additional file 1	Veitch et al. [21] Assume one hour duration of activity to convert to a MET-h
*Intervention cost estimate*		
Cost of design and construction of play-scape	$1,165,105	Personal communication Parks Victoria. 2014 costs converted to 2017 using CPI [33]
Annual maintenance	$12,700	
Life of playground	20 years	Assume 20 years (varied in sensitivity analysis)
*Control cost estimate*		
Annual maintenance	$5000	Personal communication Parks Victoria
*Sensitivity analysis*		
Difference in mean observations of children, adolescents and adults per day between T2 and T1	Additional file 1	Veitch et al. [21] Uncertainty analysis used PERT distributions of observations (Additional file 1)
Life of playground	10 and 15 years	Varied for less conservative estimates

Notes: ^*^T1: baseline, T2: first follow up at 2 months, T3: second follow up at 14 months, CPI: consumer price index. PERT: a probability distribution defined by minimum, most likely and maximum

#### Cost-effectiveness analysis

To calculate the incremental net increase in MET-h the following formula was used:

*(Intervention T3 MET-h – Intervention T1 MET-h) – (Control T3 MET-h – Control T1 MET-h)*

The costs of the play-scape were amortized over 20 years and then added to the yearly maintenance costs of $7700 (the difference between the control and intervention park maintenance costs). The net cost of the intervention was divided by the incremental net increase in MET-h. The incremental cost-effectiveness ratio was expressed as the cost per unit increase in MET-h. This is equivalent to the incremental cost per MET-h gained per park visitor.

### Sensitivity analyses

To test how robust the results were around the key assumptions, probabilistic uncertainty analysis was conducted around key parameters (Table 3). Means and 95% uncertainty intervals for yearly estimated MET-h and incremental MET-h were reported based on 2000 iterations using Ersatz version 1.35 software. Separate sensitivity analyses were conducted using combined counts of visitors observed engaging in moderate and vigorous physical activity for each age group and an average of moderate and vigorous MET-h (Table 1).

We tested the uncertainty surrounding the incremental cost-effectiveness ratio by using T2 vs T1 (instead of T3 vs T1) for the change in MET-h. Separate analysis was also conducted based on the observed visitations across 10 target areas [21] (rather than just the play-scape) using T1 vs T3, to test whether the play-scape installation had an effect on physical activity performed in other areas of the park. The life of the new play-scape was also varied using 10 and 15 years (instead of 20 years) and zero discounting of costs was modelled.

## Results

Compared with T1, a large overall increase in MET-h at T3 was observed at the intervention park, resulting in a net gain of 114,114 MET-h (95%CI =80,476 − 146,096) relative to the control park. The greatest increases in MET-h at the intervention play-scape were amongst children, with incremental gains 30 times the MET-h at baseline (Table 4). There was also a four-fold incremental increase in MET-h among adults. The average number of MET-h gained per play-scape visitor per year was 131. The change in MET-h resulted in an incremental cost-effectiveness ratio of AUD $0.58 (95% UI $0.44–$0.80) per MET-h gained, when measured against the control (Table 5).

**Table 4:** Mean MET-h at baseline and estimated incremental MET-h gained from observed play-scape visitors by age group

	Intervention park			Control park			Incremental changes in MET-h	
	Mean MET-h T1 (95% UI)	Mean MET-h T2 (95% UI)	Mean MET-h T3 (95% UI)	Mean MET-h T1 (95% UI)	Mean MET-h T2 (95% UI)	Mean MET-h T3 (95% UI)	Mean MET-h at T2* (95% UI)	Mean MET-h at T3* (95% UI)
Children (1–12 years)	2164 (1331 − 3010)	62,742 (46,374 − 80,688)	53,279 (49,417 − 84,915)	54,517 (45,153 − 64,529)	44,758 (30,276 − 58,453)	36,875 (28573–45,547)	70,581 (45645–95,479)	68,686 (48,336 − 87,845)
Adolescents (13–20 years)	0	7056 (5215 − 9074)	6318 (4617 − 7919)	1274 (1075 − 1479)	1931 (1307 − 2522)	772 (598–954)	5135 (3152 − 7267)	6820 (5125–8429)
Adults	9345 (1752 − 4146)	42,397 (31,336 − 54,524)	43,471 (31,769 − 54,486)	16,096 (13,331 − 19,052)	12,952 (8761 − 16,915)	11,584 (8976 − 14,308)	36,291 (23201–50,123)	38,638 (25,796 − 51,009)
Total	11,509 (7080 − 16,012)	112,196 (82,925 − 144,286)	102,997 (75,271 − 129,096)	71,887 (59,616 − 85,019)	59,332 (39,861 − 77,710)	49,231 (38,147 − 60,809)	113,282 (74,383–152,731)	114,114 (80,476 − 146,096)

Notes**:** UI = Uncertainty interval, T1 = baseline, T2 = 2 months after play-scape installation, T3 = 14 months after play-scape installation

Means reported from probabilistic uncertainty analysis based on 2000 iterations. *Incremental MET-h takes into account changes in visitations at the control park and changes due to the intervention

**Table 5:** Cost-effectiveness results and sensitivity analyses

Comparison time periods, area	Amortization period of play-scape	Cost-effectiveness ratio (95%UI)	Annual costs
T3 vs T1 play-scape	20 years	$0.58 ($0.44–$0.80)	$64,155
T3 vs T1 play-scape (MVPA)*	20 years	$0.56 ($0.43–$0.77)	$64,155
T2 vs T1 play-scape	20 years	$0.59 ($0.42–$0.86)	$64.155
T3 vs T1 all target areas	20 years	$0.86 ($0.24–$4.34)	$64,155
T3 vs T1 play-scape	15 years	$0.77 ($0.59–$1.06)	$85,540
T3 vs T1 play-scape	10 years	$1.15 ($0.88–$1.30)	$128,310

Notes: * used a combined moderate and vigorous physical activity category

Assuming 20 years’ amortization, the incremental annual costs of the installation and maintenance of the play-scape were $64,155.

### Sensitivity and uncertainty analyses

When moderate and vigorous activity were combined into one category (combined MVPA), the incremental gain in total estimated MET-h was slightly higher at 118,190 (95% UI: 83,528 − 149,583) resulting in a slightly lower cost per MET-h of $0.56 (95%UI: $0.43–$0.77). At T2, the incremental gain in total estimated MET-h was 113,282 (95%UI: 74,383 − 152,731) resulting in a similar cost-effectiveness ratio of $0.59 (95%UI: $0.42–$0.80) compared with T3 vs T1 (Table 5). At T2, the average number of MET-h gained per play-scape visitor per year was 118.

Reducing the amortization period from 20 years decreased the cost-effectiveness. Assuming 15 years amortization of the play-scape costs resulted in an increase in the cost-effectiveness ratio to approximately $0.77 per MET-h (95%UI: $0.59–$1.06). When amortization was further reduced to 10 years, the cost-effectiveness ratio increased to $1.15 per MET-h (95%UI: $0.88–$1.59). Reducing the discount rate from 3 to 0% had no effect on the cost-effectiveness ratios as the majority of the costs occurred in the first year. When all target areas were considered (not just the play-scape), the incremental increase in MET-h from T1 to T3 was 131,477 at a cost of $0.86 (95% UI: 0.24–4.34) per MET-h gained.

## Discussion

We estimated that 14 months after the installation of the new play-scape, the average cost per MET-h gained per person was $0.58 (95% UI $0.44–$0.80) based on amortization over 20 years. Using Wu et al’s methodology, we converted to Australian currency the cost-effectiveness benchmark of interventions targeting physical inactivity, and based on physical inactivity costing 2.4%–5% of annual healthcare costs [34] of $7096 per capita [35] and an average recommended MET-h per year of 390 MET-h. This gave a cost-effectiveness threshold ranging from approximately $0.60 to $1.30 AUD per MET-h gained (for adults). The REVAMP intervention could therefore be considered cost-effective.

The increase in MET-h measured across all ages was 113,282 (95%UI: (74,383 − 152,731) with children contributing 60% of the overall increase. Substantial increases in MET-h were also measured in adults potentially because of the increase in adults visiting the park to accompany children to the play-scape. Sensitivity analysis using an average of moderate and vigorous activity values (combined MVPA) led to more favourable results, because the MET-h values increased for moderate activity. When analysing all target areas, the results were less favourable than for the new play-scape area alone. The higher cost-effectiveness ratio reflects a larger range in the number of observed visitors and therefore more variability in the MET-h gained. Moreover, when the amortization period is reduced, the intervention becomes less cost-effective. However, local governments use a notional economic life of 20 years for playground equipment [36].

Our study compares favourably to previous studies examining the cost-effectiveness of new and refurbished parks in the US. Vacant lots converted to pocket parks with new playground equipment had cost-effectiveness ratios ranging from USD 0.43 to 2.63/MET-h [20]. Renovation of parks with new playgrounds ranged from USD 0.27 to 2.66/MET-h depending on the size of the park [14]. The cost-effectiveness of outdoor exercise equipment in parks designed for people aged 13 years and older was found to be cost-effective at USD 0.105/MET-h [17].

A review that examined the cost-effectiveness of interventions to increase physical activity, found that children’s interventions (mainly in school settings) ranged from $0.09 to $1.20 per MET-h gained (converted to AUD 2017 using purchasing power parities [37]) and our findings fall within this range [18]. The interventions analysed in that review included health and nutrition programs $0.09–$0.56 per MET-h gained [38, 39], health education plus provision of play equipment such as balls during breaks $0.68 per MET-h gained [40], and a program to encourage active transport to school $1.20 per MET-h gained [41]. Apart from the active transport program, these interventions did not involve major set up costs. The current intervention, and built environment changes in parks more generally, are long lasting and have the potential for wide population reach.

One of the strengths of the current study is the comprehensive nature of the observational data on which the analysis is based. Observations were conducted over an eight day period, four days beyond the minimum recommendation for obtaining robust measures of park visitation [42], with at least nine time points at each park per day. Strong inter-rater reliability was also achieved [21]. We also acknowledge some limitations to our study. The park observations were conducted once per hour and the count of people doing activity at a particular moment in time was used as an estimate of the total person hours of that activity during that one hour period. It is possible that the observations overestimate the amount of physical activity being undertaken, but equally may also underestimate it (e.g. visitors may have been observed sitting but were jogging for the rest of their visit). The observations are likely to be conservative as it was only possible to observe people in specified target areas and not the whole park and the observations also omitted visitors who came and left without being counted (e.g. they visited during the hour between observations). We have also assumed that the observations were representative of park visitations over a 49-week year, but it is important to acknowledge that observations conducted on other days, times and seasons may have provided different results. The study was conducted in late autumn (fall) in Melbourne, and higher numbers of visitors would be expected in spring and summer when the weather is warmer.

Further, the study findings are from one very large metropolitan park in Melbourne, Australia, which may limit generalisability of the results to other parks of varying sizes and amenities. Although the control park was located in a higher SEP area than the intervention park, the study design somewhat alleviated differences in SEP as the objective of the REVAMP study was to compare differences in changes in park visitation and park-based physical activity between the intervention and control park. The control park was located approximately 35 km from the intervention park via the road network therefore it is unlikely that visitors to the control park would have ‘migrated’ to the intervention park after the play-scape installation. However, during the observations it was not possible to determine where the visitors lived or which parks they visited previously.

Other benefits of physical activity that would increase the cost-effectiveness of the refurbishment, such as improved quality of life, mental health, social interaction, safety/injury prevention and academic performance in children [43, 44] have not been included in our study. We also did not measure the substantial long-term health care costs savings, which stem from increased physical activity [45] although this could be a topic for future research. T3 observations were conducted 14 months after the play scape installation. Future cost-effectiveness studies may benefit from a longer follow-up period.

The park refurbishment was designed specifically for children and unsurprisingly the increases in MET-h among adolescents were small. Future studies could examine the impact of designing parks to optimise physical activity among adolescents, adults and older adults. Outdoor exercise equipment in parks, for example, has been shown to be effective at increasing physical activity in adults, as well as being cost-effective [17].

## Conclusions

This study provides much needed evidence for local and state governments that the installation of a new play-scape in a metropolitan park located in an area of socioeconomic disadvantage appears to be a cost-effective intervention to facilitate greater levels of physical activity in the community. Other jurisdictions considering the installation of a new play-space in a park setting should be encouraged by these findings, which highlight the opportunity to increase park-based physical activity through investment in park refurbishment. However, replication of this study with parks of varying size, location and amenity is important, and future natural experiment studies are encouraged.

## Additional file

Additional file 1: STROBE (Strengthening The Reporting of OBservational Studies in Epidemiology) Checklist (PDF 523 kb)
